# Diagnostic Utility of Serum Ascites Lipid and Protein Gradients in Differentiation of Ascites

**DOI:** 10.1155/2019/8546010

**Published:** 2019-06-02

**Authors:** Mukhyaprana Prabhu, Rahul Sai Gangula, Weena Stanley

**Affiliations:** Department of Medicine, Kasturba Medical College, Manipal Academy of Higher Education, India

## Abstract

**Context:**

Ability of SAAG to differentiate malignant ascites from other aetiologies like tubercular peritonitis is a major problem. Alternate screening test is needed for differentiating ascites due to malignancy from those due to tubercular peritonitis.

**Aims:**

To study the diagnostic utility of serum ascites lipid gradients and serum ascites protein gradients in pathophysiological differentiation of ascites.

**Settings and Design:**

The present study is a prospective, descriptive, hospital-based, cross-sectional study.

**Methods and Material:**

The study was conducted on patients with ascites who were admitted to General Medicine Department, Kasturba Hospital, Manipal. The study included 60 patients with ascites of different etiologies (liver cirrhosis, tubercular peritonitis, and malignant ascites). All of them had undergone clinical, laboratory, and imaging investigations and were treated as per standard of care. All patients underwent abdominal paracentesis, and fluid samples were sent for analysis.

**Statistical Analysis Used:**

ANOVA, Kruskal-Wallis H test, and ROC curve analysis.

**Results:**

Among the gradients, only SAPG and SAAG had over all statistical significance (<0.005) among the groups, but no significance between malignancy and tubercular peritonitis had been observed. Similarly all the ascitic fluid parameters measured had an overall statistical significance (<0.005), but there was no significant difference observed between malignancy and tubercular peritonitis groups. However, ascitic fluid and serum HDL cholesterol had a statistical significance (<0.05) between malignancy and tubercular peritonitis.

**Conclusions:**

With a cut-off value of 4, SAPG is one of best screening tests in differentiation of cirrhotic with noncirrhotic ascites when compared with SAAG, whereas it is a poor parameter with high sensitivity and very low specificity in differentiation of malignant with nonmalignant ascites. Also the present study reveals HDL cholesterol levels in ascitic fluid to be a valuable marker with higher sensitivity and specificity in differentiation of malignancy and tuberculosis peritonitis (i.e., differentiation of low SAAG ascites).

## 1. Introduction

Ascites is the pathological accumulation of fluid in peritoneal cavity [1, 2]. Most common causes of ascites are parenchymal liver disease followed by peritoneal malignancy, tubercular peritonitis, congestive cardiac failure, nephrotic syndrome, and others (hypoalbuminemia, chylous ascites, Budd-Chiari syndrome, mixed ascites, and malnutrition) [1, 2]. Treatment of ascites depends on the aetiology of ascites, for which numerical diagnostic parameters were investigated [3, 4]. However no single parameter has completely demarcated among them; thus the quest for better investigation continues [3, 4].

According to the traditional way of classification, aetiology of ascites were differentiated based on transudate and exudate concept with levels of Total Protein (> 2.5 gm/dL) in ascitic fluid [5–7]. However SAAG criteria had completely replaced the traditional way of classification [7]. According to SAAG criteria, the Serum Ascites Albumin Gradient ≥ 1.1 gm/dL is usually associated with increased portal pressure [7]. But the ability of SAAG to differentiate malignant ascites from other etiologies is a major problem [7].

Ascitic fluid cytology is considered as standard test for malignancy but its sensitivity is low [8]. Both tubercular peritonitis and malignant ascites have similar presentation (both are chronic) and parameters tested (including tumour markers) have overlapping results [9–13]. It is mandatory to differentiate them at an early stage as the treatment diverges. Early diagnosis could improve the morbidity and mortality associated. So, alternate screening test is needed for differentiating ascites due to malignancy from that due to tubercular peritonitits.

Some of recent studies had showed higher diagnostic significance of Ascitic Fluid Cholesterol and Serum Ascites Cholesterol Gradient in ascites due to malignancy [14–17]. The present study was done to reveal the diagnostic utility of serum ascites lipid gradients in patients with ascites and compared it with standard SAAG criteria, to know whether it has a higher diagnostic yield. Also the present study aims to determine whether serum ascites lipid gradients can differentiate between malignant and tubercular peritonitis ascites.

### 1.1. Objectives

To study the diagnostic utility of SALG and SAPG in differentiation of ascites and compare them with SAAG.

To study the diagnostic utility of Ascitic Fluid Cholesterol and Total Protein in differentiation of ascites.

## 2. Subjects and Methods

We did this hospital based, prospective, descriptive, cross-sectional study in our tertiary care hospital located in Manipal, Karnataka. The study was conducted after obtaining approval from Institutional Ethics Committee (IEC 625/2016). The study had considered patients who were admitted in general medicine department between September 2016 and September 2018 with abdominal distension due to ascites and were screened for inclusion into the study.

### 2.1. Sample Size

Sample size had been calculated before starting the study, based on comparison of means formula with a 95% confidence interval and power of 80%. Sample size for each group was calculated to 20 and total sample size of the study was calculated to 60 (20 x 3).

### 2.2. Informed Consent

Informed consent was obtained from all the individuals prior to inclusion into the study group.

### 2.3. Criteria to Group, Definition, and Classification

Patients whose clinical, biochemical, and radiological investigations were suggestive of chronic liver disease and ultrasound showing coarse echotexture with surface nodularity of liver were included into cirrhosis group.

Patients whose ascitic fluid malignant cytology (or) histopathological evidence of tissue from peritoneum suggestive of malignancy were included in malignancy group.

Patients whose peritoneal biopsy Gene Xpert (or) PCR for Mycobacterium tuberculosis is positive were included in tubercular peritonitis group.

### 2.4. Inclusion and Exclusion Criteria

Patients who had mixed ascites (cirrhosis with peritoneal malignancy and cirrhosis with peritoneal tuberculosis) were also included in the study. Patients who were found to have ascites due to Budd-Chiari syndrome, nephrotic syndrome, or chylous ascites were excluded.

### 2.5. Sample Collection Methodology

All the patients included in the study had undergone detailed clinical examination at the time of admission. All the patients were subjected to routine laboratory investigations and standard care for diagnosis of ascites. In all the patients who were included in the study, a right sided abdominal paracentesis was done in the fasting state (fasting of minimum 6 hours) and ascitic fluid was collected and was analyzed for total proteins, albumin, lipid profile, and other routine investigations. Simultaneous blood samples were collected for analyzing serum total proteins, serum albumin, lipid profile, and other routine investigations. Supportive laboratory tests like diagnostic laparoscopy for peritoneal biopsy histopathology, ascitic fluid ADA (Adenosine De Aminase) levels, and ascitic fluid cytology were performed when needed.

### 2.6. Study Variables, Outcome Measures, and Unit of Measurement

Gradient of each variable is calculated by the following formula:

Serum ascites ‘X' gradient = ‘X' concentration in serum – ‘X' concentration in ascitic fluid

where ‘X' refers to the substance of interest.

### 2.7. Statistical Analysis/Data Analysis

Data were collected into predesigned proforma and were entered into Microsoft Office Professional Plus Excel 2013 (Microsoft Corp, Redmond, USA). The entry of data was cross-checked at two levels (entry into proforma, entry from proforma to Excel sheet) by two independent observers, to avoid any possible error in entry. Descriptive statistics for the categorical variables were performed by computing the frequencies (percentages) in each category. Type of distribution of the variables was checked using Shapiro-Wilk test of normality. P value of < 0.05 in Shapiro-Wilk test of normality was considered as significant and the distribution was taken as non-Gaussian distribution. The quantitative variables, which had normal distribution, were summarized by mean and standard deviation. The quantitative variables, which had skewed distribution, were summarized by median and interquartile range. For variables with Gaussian distribution, one-way analysis of variance (ANOVA) was used to compare means among the groups, whereas for variables with non-Gaussian distribution, Kruskal-Wallis H test was used to compare medians among the groups. P value < 0.05 was taken as statistically significant. Receiver Operating Characteristic (ROC) Curve was used to determine Area Under the Curve (AUC), whereas Plot vs. Criterion value graphs were used to define the optimal cut-off value for each variable, to differentiate study population into two groups based on etiology of ascites. Cut-off values are calculated for highest Youden index (sensitivity + specificity) for each variable. Statistical analysis was performed using Statistical Package for the Social Sciences (SPSS) Statistics, Version 20 (IBM SPSS Statistics, Somers NY, USA) and MedCalc Version 18.5.0 for 32-bit Windows 7 Enterprise (MedCalc Statistical Software, Belgium). All the values in the mean, median, standard deviation, and interquartile range are taken into consideration till accuracy of two decimal points. Cut-off values and p values till 1 decimal point and 3 decimal points were calculated, respectively.

## 3. Results

During the study a total of 60 patients were included into study. Baseline characteristics (laboratory parameters) among the groups were not statistically significant (Table 1).

Overall statistical significance of parameters for gradient (total protein, albumin) was observed between the groups. However none of the gradients had any statistical difference between malignancy and tubercular peritonitis groups (Table 2).

Similar to gradients, ascitic fluid levels of protein, albumin, and lipids were overall statistically significant among groups. But there was no difference observed between malignancy and tubercular peritonitis groups for the same variables. However the present study had showed that HDL cholesterol levels in ascitic fluid were statistically significant (p=0.006) between malignancy and tubercular peritonitis groups.

**Figure 1:**
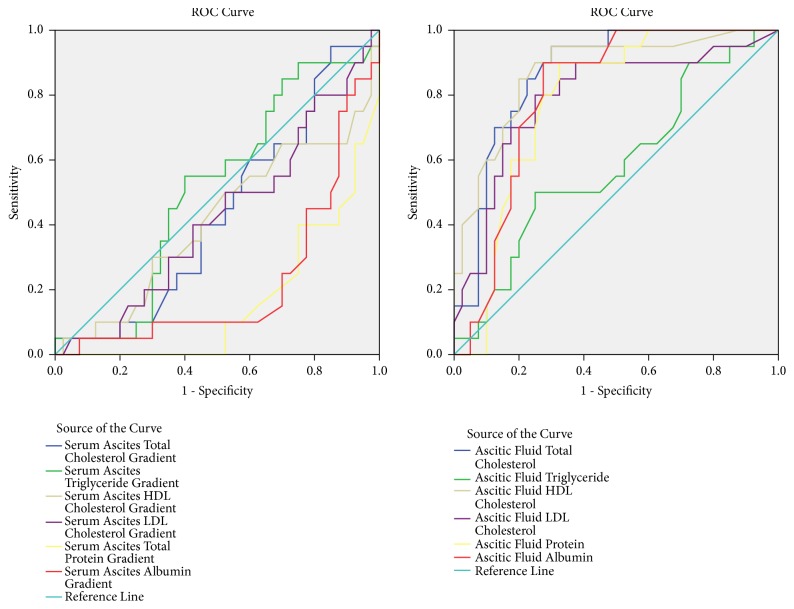
ROC curve analysis between malignant vs. nonmalignant ascites.

**Figure 2:**
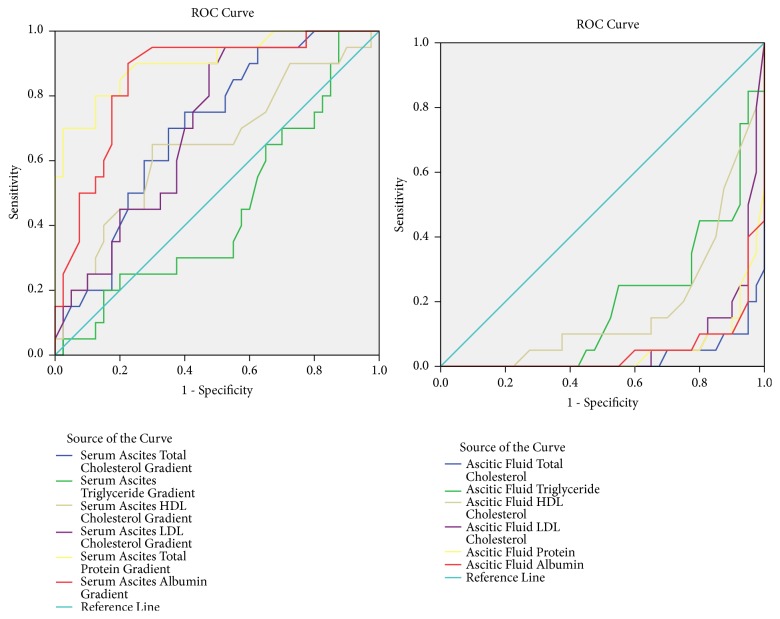
ROC curve analysis between cirrhotic vs. noncirrhotic ascites.

ROC curve analysis of variables in differentiating malignant from nonmalignant ascites and cirrhotic from noncirrhotic ascites was shown in the graphs and table (Figures 1 and 2; Table 3).

Cut-off values with sensitivity and specificity for each variable in differentiating malignant from nonmalignant ascites and cirrhotic from noncirrhotic ascites were calculated (Table 4).

## 4. Discussion

In the present study we have evaluated lipid and total protein levels in serum and ascites for differentiation of ascites. The present study is unique in nature in considering serum ascites total protein gradient for differentiation of ascites. Also the study is one of the few which had considered including mixed ascites to study.

In the present study, the mean age at presentation among the three groups showed higher mean age for malignancy group. Cases in cirrhosis were uniformly distributed over the middle aged and elderly, while malignancy was more present in the elderly. Tubercular peritonitis group had most cases in the middle aged. Distribution of cases could be imagined as rectangle, inverted triangle, and rhomboid shapes in cirrhosis, malignancy, and tubercular peritonitis groups, respectively. As the present study consists predominantly of alcohol related cirrhosis, almost 90 percent of the group are males. Malignancy group in the present study had showed unequal distribution with higher female cases. The most common primary tumour among them is ovarian carcinoma. The present study also had higher male cases in tubercular peritonitis group. This could be due to inclusion of mixed ascites in the study. As cirrhosis is one of the risk factors, and the present study had higher alcohol related cirrhosis, the above distribution among the gender was expected.

### 4.1. Lipid Gradients

In the present study, to differentiate cirrhotic ascites from noncirrhotic ascites, lipid gradients (TC, TG, HDL, and LDL) had sensitivities and specificities of 75%, 70%, 65%, 90% and 60%, 55%, 70%, 52.5% with cut-off values of 69 mg/dL, 61 mg/dL, 11 mg/dL, and 29 mg/dL, respectively.

In a study conducted by Sharathchandra et al. [18], lipid gradients (TC, TG, HDL, and LDL) had sensitivities and specificities of 80%, 52%, 60%, 76% and 80%, 52%, 60%, 76% with cut-off values of 65 mg/dL, 65 mg/dL, 27 mg/dL, and 47 mg/dL, respectively.

Similar study conducted by Morsy et al. [19] found lipid gradients (TC, TG, HDL, and LDL) had sensitivities and specificities of 90%, 65%, 65%, 82% and 92%, 60%, 70%, and 75% with a cut-off values of 67 mg/dL, 66 mg/dL, 26 mg/dL, and 49 mg/dL, respectively.

Also a study conducted by Ranjith et al. [20] showed lipid gradients (TC, TG, HDL, and LDL) that had sensitivities and specificities of 93.3%, 83.3%, 90%, 86.7% and 90.3%, 96.6%, 90%, 90% with cut-off values of 63.5 mg/dL, 63.5 mg/dL, 19.75 mg/dL, and 36 mg/dL, respectively. This study had also included noncirrhotic portal hypertension patients.

Also the present study showed that, to differentiate the malignant ascites from the nonmalignant ascites lipid gradients (TC, TG, HDL, and LDL) had sensitivities and specificities of 75%, 90%, 35%, 50% and 45%, 25%, 90%, 67.5% with cut-off values of 82 mg/dL, 34 mg/dL, 1 mg/dL, and 29 mg/dL, respectively. In previous similar studies conducted by Sharathchandra et al. [18], Morsy et al. [19], and Ranjith et al. [20] cut-off values, sensitivities, and specificities for all lipid gradients used for differentiating the malignant ascites from the nonmalignant ascites were not calculated. Hence our results could not be compared as data are lacking.

However the previous studies had calculated only serum ascites cholesterol gradient. Vyakaranam et al. [15] in their study had showed that SACG had sensitivity of 90% and specificity of 95% at a cut-off value of < 53 mg/dL, in differentiating malignant from nonmalignant ascites, whereas a similar study by Dharwadkar et.al [21] showed that SACG had sensitivity of 68% and specificity of 100% at a cut-off value of < 95 mg/dL, in differentiating cirrhotic from tubercular peritonitis ascites.

### 4.2. Protein Gradients

In the present study, SAPG had sensitivity of 100% and specificity of 52.5% in differentiating malignant from nonmalignant ascites, at a cut-off value of 4 gm/dL, whereas at a cut-off value of >4 gm/dL, SAPG had sensitivity of 80% and specificity of 87.5% in differentiating cirrhotic from noncirrhotic ascites. None of the previous studies had evaluated protein gradient in differentiation of ascites; hence data is lacking for comparison.

However the present study had showed that SAAG had sensitivity of 90% and specificity of 77.5% in differentiating cirrhotic from noncirrhotic ascites at a cut-off value of > 1.1 gm/dL, whereas it had sensitivity of 85% and specificity of 70% in differentiation of malignant from nonmalignant ascites at a cut-off value of 1.08 gm/dL.

Similar studies conducted by Vyakaranam et al. [15] and Gupta et al. [22] showed that SAAG had sensitivities of 96%, 94% and specificities of 92%, 91%, respectively, at a cut-off value of 1.1 gm/dL, in differentiating cirrhotic from noncirrhotic ascites.

### 4.3. Ascitic Fluid Lipids

In the present study, to differentiate cirrhotic from noncirrhotic ascites, ascitic fluid lipid levels (TC, TG, HDL, and LDL) had sensitivities and specificities of 90%, 75%, 80%, 85% and 95%, 77.5%, 75%, 90% with a cut-off values of 29 mg/dL, 39 mg/dL, 6 mg/dL, and 9 mg/dL, respectively.

A study conducted by Sharathchandra et al. [18] showed ascitic fluid lipid levels (TC, TG, HDL, and LDL) had sensitivities and specificities of 96%, 76%, 78%, 88% and 96%, 88%, 78%, 88% with cut-off values of 67 mg/dL, 40 mg/dL, 9.1 mg/dL, and 35 mg/dL, respectively. A similar study by Gupta et al. [22] had showed ascitic fluid cholesterol had sensitivity of 94% and specificity of 94% at a cut-off value of 55 mg/dL.

Also in the present study, to differentiate malignant from nonmalignant ascites, ascitic fluid lipid levels (TC, TG, HDL, and LDL) had sensitivities and specificities of 95%, 50%, 90%, 80% and 70%, 75%, 75%, 75% with cut-off values of 51 mg/dL, 49 mg/dL,11 mg/dL, and 34 mg/dL, respectively.

Many previous studies had only evaluated ascitic fluid cholesterol levels in differentiation of ascites. Studies by Sastry et al. [23] and Rana et al. [24], in differentiating malignant from nonmalignant ascites, showed that ascitic fluid cholesterol had sensitivities of 90%, 88% and specificities of 97.5%, 100% at cut-off values of > 62 mg/dL, > 70 mg/dL respectively.

A study by Sood et al. [25] showed that at a cut-off value of > 54.5 mg/dL, ascitic fluid cholesterol had sensitivity of 89.65% and specificity of 100% in differentiating malignant from tubercular peritonitis ascites, whereas a study by Cabral et.al [16] showed that at a cut-off value of > 48 mg/dL, ascitic fluid cholesterol had sensitivity of 82.4% and specificity of 85.4% in differentiating malignant from cirrhotic ascites. A study by Dharwadkar et al. [21] showed that at a cut-off value of > 70 mg/dL, ascitic fluid cholesterol had sensitivity of 100% and specificity of 95.5% in differentiating tubercular peritonitis from cirrhosis ascites.

### 4.4. Ascitic Fluid Proteins

In the present study, ascitic fluid protein had sensitivity of 90% and specificity of 67.5% in differentiating malignant from nonmalignant ascites, at a cut-off value of > 3.3 gm/dL, whereas at a cut-off value of 2 gm/dL, it had sensitivity of 90% and specificity of 90% in differentiating cirrhotic from noncirrhotic ascites.

Also the present study showed that ascitic fluid albumin levels had sensitivity of 90% and specificity of 72.5% in differentiating malignant from nonmalignant ascites, at a cut-off value of > 1.7 gm/dL, whereas at a cut-off value of 0.8 gm/dL, it had sensitivity of 90% and specificity of 90% in differentiating cirrhotic from noncirrhotic ascites.

A study conducted by Gupta et al. [22] in differentiating cirrhotic from noncirrhotic ascites had showed that ascitic fluid protein at cut-off value of 2.5 gm/dL had sensitivity of 76% and specificity of 100%, whereas ascitic fluid albumin at a cut-off value of 2 gm/dL had sensitivity of 82% and specificity of 100%.

A study by Rana et al. [24] in differentiation of malignant from nonmalignant ascites showed that ascitic fluid total protein at a cut-off value of >3gm/dL had a sensitivity of 56% and specificity of 88%.

### 4.5. Summary

We observed a statistically significant difference among the groups for SAPG and SAAG and ascitic fluid protein, albumin, and lipid levels. However, only HDL cholesterol levels had significant difference between malignant and tubercular peritonitis groups. These results were in contrast with previous studies. The possible explanation for the discrepancy might be due to inclusion of mixed ascites. Cirrhosis is one of the risk factors for peritoneal tuberculosis. Exclusion of mixed ascites (cirrhosis with peritoneal tuberculosis) in previous studies might have led to insignificant difference.

Though the present study had revealed higher sensitivity and specificity for ascitic fluid protein and lipid levels, the levels in ascitic fluid tend to alter with patient on a treatment like diuretics [21, 26]. However similar discrepancy is not observed with gradients as confirmed by previous studies [18–20, 27].

Also the cut-off values obtained in the present study for ascitic fluid lipids and protein were not similar to previous studies [16, 17, 21], whereas the cut-off value for SAAG was reliable, as confirmed by previous studies [18–20, 27].

### 4.6. Underlying Mechanism

Accumulation of fluid in peritoneal fluid might be due to different pathogenic mechanisms. In cirrhosis, fluid accumulation is transudative and due to altered starling forces. The permeability of peritoneal membrane is not altered when compared to normal individuals, whereas both malignancy and tubercular peritonitis are exudative and are due to increased permeability of peritoneal membrane.

Inflammation of peritoneum leads to permeation of the membrane by various solutes. Permeability is dependent on thickness, pore size, and the charge over the membrane. Though the albumin is smaller than the pore between podocytes of glomerular membrane in a normal kidney, it is not permeable due to the charge over the membrane, whereas it traverses through membrane in patients of nephrotic syndrome due to alteration of membrane surface charge or opening of larger pores. Similar pathophysiology might help us understand better the difference between malignancy and tubercular peritonitis ascitic fluid accumulation.

Previous studies conducted in patients on peritoneal dialysis formulated a “three-pore model” for the mechanism of transport of solute through peritoneum [28]. According to the study, peritoneum consists of very small pores, small pores and large pores [28]. The very small pores are helpful for transcellular transport whereas small and large pores are helpful for paracellular transport of solutes [28]. Paracellular transport through small and large pores depends on solute size, glycocalyx over the peritoneal membrane, and intercellular fibres between cells lining peritoneal membrane [28].

Peritoneal inflammation causing increased permeability might be due to loss of glycocalyx and opening of large pores [28], whereas in peritoneal malignancy it might be due to opening of large pores, loss of intercellular fibres, and cells actively secreting into peritoneal fluid due to metastases. Though HDL lipoproteins are slightly larger than the albumin and gamma globulins, they are not increased in peritoneal inflammation [29, 30]. This might be due to the unique composite molecular structure which has both lipids and proteins [29, 30]. However it also depends on the overall charge carried by the lipoprotein surface which depends on fraction of surface proteins [29]. This could possibly explain the statistical difference of HDL cholesterol levels in ascitic fluid between malignancy and tuberculosis observed in the present study.

## 5. Conclusion

Lipid gradients are not better indicators for differentiation when compared with SAAG; however SAPG has higher yield in differentiation of ascites. Though the present study could establish superiority of SAPG, previous data is lacking to confirm this. Also the present study showed that HDL cholesterol levels might help in differentiation of malignancy from tubercular peritonitis. Further studies are required with a larger sample size to validate the results. Till then SAAG continues to be the best parameter in differentiation of ascites (high vs. low), but it lacks differentiation of low SAAG ascites.

## Figures and Tables

**Table 1:** Baseline characteristics of patients in three groups.

Characteristics	Cirrhosis (n=20)	Malignancy (n=20)	Tuberculosis (n=20)
Hemoglobin (gm/dL)	9.59 ± 1.83	11.00 ± 2.22	10.17 ± 2.36

WBC Count (x 103 *μ*L)	9.54 ± 4.87	9.4 ± 2.37	9.69 ± 6.57

Platelet Count (x 103 *μ*L)	156.05 ± 95.75	392.44 ± 210.54	272.35 ± 154.04

Total Bilirubin (mg/dL)	5.34 ± 6.74	0.70 ± 0.88	1.94 ± 2.56

Serum Total Protein (gm/dL)	6.48 ± 1.30	6.42 ± 0.65	6.66 ± 1.21

Serum Albumin (gm/dL)	2.28 ± 0.59	3.28 ± 0.45	3.00 ± 0.92

Serum AST (IU/L)	68.45 ± 31.35	33.40 ± 28.05	59.8 ± 55.29

Serum ALT (IU/L)	32.75 ± 24.07	20.65 ± 30.94	28.05 ± 34.61

Serum ALP (IU/L)	197.90 ± 226.62	180.75 ± 188.18	172.5 ± 144.71

Serum Urea (mg/dL)	28.95 ± 22.25	22.60 ± 15.05	57.95 ± 67.04

Serum Creatinine (mg/dL)	1.34 ± 1.25	0.79 ± 0.16	2.40 ± 2.90

Serum Sodium (mmol/L)	132.05 ± 4.92	134.55 ± 4.71	131.8 ± 6.95

Serum Potassium (mmol/L)	4.03 ± 0.68	4.34 ± 0.59	4.65 ± 0.63

Prothrombin Time (seconds)	17.05 ± 4.01	11.57 ± 1.28	13.21 ± 2.25

INR	1.59 ± 0.38	1.08 ± 0.14	1.23 ± 0.23

**Table 2:** Serum, Ascitic Fluid and Serum Ascites Gradients: Total Protein, Albumin, Total Cholesterol, Triglycerides, HDL Cholesterol, and LDL Cholesterol in the three study groups.

Parameters		Cirrhosis		Malignancy		Tubercular		P value			
		MEAN	MEDIAN	MEAN	MEDIAN	MEAN	MEDIAN	Overall	C Vs M	C Vs T	M Vs T
		S.D	I.Q.RANGE	S.D	I.Q.RANGE	S.D	I.Q.RANGE				
Serum	Total Protein	6.48		6.42		6.67		0.731	0.981	0.859	0.757
		1.30		0.65		1.21					
	Albumin	2.28		3.28		3.00		**0.001**	**0.001**	**0.004**	0.397
		0.58		0.45		0.92					
	Total Cholesterol	111.00		161.10		124.90		**0.001**	**0.001**	0.925	**0.032**
		36.85		35.08		47.56					
	Triglyceride	113.60	83.67	140.05	121.5	149.20	125.00	0.135	NS	NS	NS
		81.621	69.50-132.00	95.27	91.67-148.00	96.58	75.67-199.50				
	HDL Cholesterol	21.80	18.67	31.45	31.50	19.90	19.00	**0.006**	**0.028**	1.000	**0.012**
		16.32	7.67-32	9.82	24.75-38.25	13.63	9.00-25.67				
	LDL Cholesterol	66.60	60.67	95.40	88.50	75.20	71.33	**0.024**	**0.024**	0.699	0.148
		32.65	40.00-84.00	31.74	78.33-123	36.34	47.50-103.50				

Ascitic Fluid	Total Protein	1.29	1.08	4.35	4.75	3.93	4.05	**0.001**	**0.001**	**0.001**	1.000
		0.84	0.77-1.53	1.06	4.00-5.05	1.64	2.65-5.05				
	Albumin	0.50	0.36	2.40	2.60	1.93	2.00	**0.001**	**0.001**	**0.001**	0.743
		0.49	0.24-0.54	0.64	2.15-2.82	0.96	1.15-2.80				
	Total Cholesterol	17.75	12.80	93.15	92.00	63.15	56.50	**0.001**	**0.001**	**0.001**	0.163
		12.54	11.00-17.50	37.51	64.50-104.00	28.78	46.25-82.50				
	Triglyceride	32.40	31.00	70.40	46.50	63.15	48.50	**0.001**	**0.015**	**0.001**	0.984
		12.52	23.67-43.00	97.10	34.67-64.50	34.13	42.33-74.50				
	HDL Cholesterol	5.35	2.88	23.50	22.50	10.25	8.00	**0.001**	**0.001**	0.168	**0.006**
		6.45	1.67-5.67	13.34	15.67-27.50	8.02	3.50-16.00				
	LDL Cholesterol	6.10	3.40	57.65	62.00	40.25	35.00	**0.001**	**0.001**	**0.001**	0.773
		8.49	1.00-7.00	31.34	36.00-77.50	26.08	18.00-62.00				

Serum Ascites Gradient	Total Protein	5.19		2.05		2.84		**0.001**	**0.001**	**0.002**	0.151
		1.68		0.78		1.17					
	Albumin	1.78	1.78	0.88	0.85	1.12	1.03	**0.001**	**0.001**	**0.004**	0.481
		0.54	1.43-2.14	0.51	0.69-1.02	0.50	0.87-1.46				
	Total Cholesterol	93.15	94.00	67.95	64.00	61.75	48.50	0.056	0.181	0.074	0.898
		38.31	62.50-115.00	46.89	37.50-89.50	47.53	23.50-106.50				
	Triglyceride	81.20	55.00	69.70	67.50	85.95	66.00	0.801	NS	NS	NS
		78.02	37.00-103.00	133.36	47.33-91.00	85.15	36.50-154.00				
	HDL Cholesterol	16.65	14.67	7.95	7.67	9.65	7.00	0.175	NS	NS	NS
		15.97	4.67-24.5	16.97	(-1.00)-17.33	9.34	3.33-13.20				
	LDL Cholesterol	60.55	50.00	37.85	34.00	34.80	26.00	**0.040**	0.094	**0.050**	0.956
		32.22	36.00-78.5	32.20	16.00-60.00	36.88	9.00-58.50				

**Table 3:** Area under the ROC curve analysis for serum ascites gradients and ascitic fluid: Total protein, Albumin, Total Cholesterol, Triglycerides, HDL Cholesterol, LDL Cholesterol between Malignant vs. Nonmalignant groups and Cirrhotic vs. Noncirhhotic groups.

Parameter	Malignant vs. Nonmalignant				Cirrhotic vs. Noncirrhotic			
	Area Under Curve	Asymptotic Significance	Asymptotic 95% Confidence Interval		Area Under Curve	Asymptotic Significance	Asymptotic 95% Confidence Interval	
			Lower Bound	Upper Bound			Lower Bound	Upper Bound
Serum Ascites Total Protein Gradient	0.163	**0.001**	0.063	0.262	0.905	**0.001**	0.820	0.990
Serum Ascites Albumin Gradient	0.224	**0.001**	0.096	0.351	0.859	**0.001**	0.758	0.959
Serum Ascites Total Cholesterol Gradient	0.434	0.410	0.285	0.584	0.699	**0.013**	0.565	0.832
Serum Ascites Triglyceride Gradient	0.509	0.906	0.358	0.661	0.450	0.530	0.295	0.605
Serum Ascites HDL Cholesterol Gradient	0.414	0.279	0.254	0.574	0.648	0.063	0.494	0.802
Serum Ascites LDL Cholesterol Gradient	0.425	0.347	0.272	0.578	0.705	**0.010**	0.574	0.836

Ascitic Fluid Total Protein	0.782	**0.001**	0.665	0.898	0.049	**0.001**	0.000	0.099
Ascitic Fluid Albumin	0.804	**0.001**	0.694	0.914	0.051	**0.001**	0.000	0.105
Ascitic Fluid Total Cholesterol	0.868	**0.001**	0.778	0.958	0.029	**0.001**	0.000	0.067
Ascitic Fluid Triglyceride	0.584	0.290	0.430	0.739	0.196	**0.001**	0.084	0.309
Ascitic Fluid HDL Cholesterol	0.873	**0.001**	0.777	0.969	0.182	**0.001**	0.070	0.294
Ascitic Fluid LDL Cholesterol	0.794	**0.001**	0.666	0.922	0.069	**0.001**	0.005	0.133

**Table 4:** Cut-off values for serum ascites gradients and ascitic fluid: Total protein, Albumin, Total Cholesterol, Triglycerides, HDL Cholesterol, and LDL Cholesterol between Malignant vs. Nonmalignant groups and Cirrhotic vs. Noncirrhotic groups.

Parameter	Malignant vs. Nonmalignant			Cirrhotic vs. Noncirrhotic		
	Cut-off value	Sensitivity	Specificity	Cut-off value	Sensitivity	Specificity
Serum Ascites Total Protein Gradient	≤ 4	100	52.5	> 4	80	87.5
Serum Ascites Albumin Gradient	≤ 1.08	85	70	> 1.1	90	77.5
Serum Ascites Total Cholesterol Gradient	≤ 82	75	45	> 69	75	60
Serum Ascites Triglyceride Gradient	> 34	90	25	≤ 61	70	55
Serum Ascites HDL Cholesterol Gradient	≤ 1	35	90	> 11	65	70
Serum Ascites LDL Cholesterol Gradient	≤ 29	50	67.5	> 29	90	52.5

Ascitic Fluid Total Protein	> 3.3	90	67.5	≤ 2	90	90
Ascitic Fluid Albumin	> 1.7	90	72.5	≤ 0.8	90	90
Ascitic Fluid Total Cholesterol	> 51	95	70	≤ 29	90	95
Ascitic Fluid Triglyceride	> 49	50	75	≤ 39	75	77.5
Ascitic Fluid HDL Cholesterol	> 11	90	75	≤ 6	80	75
Ascitic Fluid LDL Cholesterol	> 34	80	75	≤ 9	85	90

## Data Availability

The data used to support the findings of this study are available from the corresponding author upon request.
